# A Deflationary Account of Information in Terms of Probability

**DOI:** 10.3390/e27050514

**Published:** 2025-05-11

**Authors:** Riccardo Manzotti

**Affiliations:** Department of Business, Law, Economics and Behaviour, Faculty of Communication, IULM University, 20143 Milano, Italy; riccardo.manzotti@gmail.com

**Keywords:** information, Ockham razor, probability, causation, Eleaticism, philosophy of science, theory of information, ontology

## Abstract

In this paper, I argue that information is nothing more than an abstract object; therefore, it does not exist fundamentally. It is neither a concrete physical entity nor a form of “stuff” that “flows” through communication channels or that is “carried” by vehicles or that is stored in memories, messages, books, or brains—these are misleading metaphors. To support this thesis, I adopt three different approaches. First, I present a series of concrete cases that challenge our commonsensical belief that information is a real entity. Second, I apply Eleaticism (the principle that entities lacking causal efficacy do not exist). Finally, I provide a mathematical derivation showing that information reduces to probability and is therefore unnecessary both ontologically and epistemically. In conclusion, I maintain that information is a causally redundant epistemic construct that does not exist fundamentally, regardless of its remarkable epistemic convenience. What, then, is information? It is merely a very efficient way of describing reality—a manner of speaking, nothing more.

## 1. Does Information Exist?

Why ask this apparently outdated question that, to be fair, has been already asked many times [1,2,3]? More than 70 years since Claude Shannon’s foundational article, where the issue of mathematically quantifying information was addressed for the first time [4], should it not be clear to everyone what information is? Unfortunately not. As we will see, the notion of information as something real or physical, or even as a substance that is created, carried, transferred, and stored, is mostly a misleading metaphor.

This paper defends a radical thesis: while epistemically useful and practically convenient, the concept of information is ontologically and causally insignificant, as well as mathematically redundant. While such a notion serves as a convenient simplification, it should not be regarded as a fundamental constituent of physical reality. Information is akin to abstract objects, such as centers of mass or calories (more on both soon) [5].

Is the effort to debunk the notion of information justified? Or is it a strawman argument? The main motivation for this effort is given by the widespread tendency in many fields—from biology to philosophy of mind, from semantics to AI—to construct a conceptual house of cards based on the assumption that information is a physical entity. In contrast, if information were regarded only an abstract concept, such models would collapse. This is particularly effective in the field of computational models of the mind or in that of consciousness studies, where a deflationary approach to information would compel many theorists to reconsider their models in a more ontologically parsimonious way.

Many consider information a basic constituent of reality, akin to a fundamental attribute of the physical world. It is an attitude akin to what the philosopher of physics Christopher G. Timpson christened *informational immaterialism*, namely “the thought that information is perhaps the fundamental category from which all else flows (a view with obvious affinities to idealism)” [6] (p. 1). For example, statements such as, “information is a physical entity that can be generated, transmitted, and received for practical purposes” are common and almost never criticized [2]. For laypeople, information is generated, transmitted, stored. To a large extent, the concept of information has been reified. In science, many physicists and most engineers deal with information as though it were a physical entity “generated at one point of the physical space and transmitted to another point; accumulated, stored, and converted from one form to another” [7]. For example, it is common to read that “the light entering the eye carries information about the relative distances of objects” [8], that “it is widely recognized that DNA carries information” [9], or that “the brain processes information” [10], let alone the concept of computers as devices capable of storing, processing, and transmitting information.

It is no coincidence that in the field of theoretical physics, some scientists have gone even further, even arguing that information is the primary substance from which everything is derived. John Wheeler’s “It from bit” has become a popular catchphrase, “every it—every particle, every field of force, even the spacetime continuum itself—derives […] its very existence entirely […] from the apparatus elicited answers to yes-or-no questions, binary choices […], bits” [11]. Many scientists trust Landauer’s popular claim that “information is physical” [12,13,14]. Or, finally, one may be fascinated by Tononi’s claim that integrated information is even more fundamental than other familiar physical quantities: “If we were able to measure integrated information—instead of mass, electrical charges, and energy—the planets and the galaxies would turn to grey dust and the universe would become even emptier and colder” [15].

The main concern of this paper is the common tendency to attribute an unjustified ontological status to information. In what follows, I will advocate for a probability-based, deflationary interpretation of Shannon’s formula, which requires no additional ontological commitments. A caveat is that I will focus only on the classic Shannon formula (thereby leaving for a future paper other measures of information such as Rényi entropy, Tsali’s entropy, intrinsic information, or von Neumann entropy). This focus is motivated by the importance that Shannon’s formula has had in shaping the information age and the way in which scholars and laypeople generally think about information. The most recent measures cannot compete with Shannon’s in term of influence and popularity.

Of course, information would be a useful tool regardless its ontological status. As an epistemic construct, information is undeniably useful, since, as the philosopher Arnon Levy observed, “scientists employ a range of reasoning strategies that involve imagining away some elements of reality or pretending that things are different than they actually are: idealizations, simplifications, approximations” [16]. The probability-based interpretation I propose does not diminish the practical usefulness of information; instead, it clarifies its ontological status and illustrates why it is such a pervasive concept. This paper does not challenge the utility of the mathematical framework of information, nor does it question the validity of analogies drawn from other fields in which statistical properties play crucial roles. Rather, my critique targets the notion that information is a substance or entity existing over and above the physical events constituting reality.

Many authors distinguish between information and pieces of information. For instance, Timpson states that, according to Shannon, “the task is to reproduce at one point, either exactly or approximately, a message selected at another. That is, to reproduce at a far point whatever it was that the information source produced. The pieces of information to be transmitted, then, are simply what it is that is produced by the source.” [6] (p. 16). I cannot agree more—the pieces or vehicles of information are surely physical entities. Likewise, both Shannon’s noiseless coding theorem and Kolmogorov’s complexity quantitatively estimate the minimum number of abstract operations required for a message to be accurately reconstructed at the other end of a channel. It is customary to distinguish between information as quantified by Shannon’s or Kolmogorov’s measures and the pieces of data that serve as the physical carriers of these operations. Nonetheless, it is worth considering whether these should be referred to as pieces of information or rather as physical intermediate elements in a causal process with specific statistical properties, as described by Shannon and Kolmogorov. This question of whether the term “information” is necessary at all remains. Is the noun “information” anything but an epistemic construct? Claiming that a PDF or a printed letter is a piece of information is no different from saying that slipping on a banana peel is a *piece of bad luck*. In the latter cases, no ontological status is being attributed to bad luck. Does information exist? Many authors speak of information as if it were some concrete entity; however, if we scratch beneath the surface, we discover that there are unsettling doubts. Consider the simplest question: have you ever directly observed information itself? One quickly realizes there is something peculiar about information, despite its apparent simplicity and ubiquity, namely that no one has ever encountered information directly. Who has ever seen a bit? We perceive images or texts allegedly formed by information, yet who has observed information as information? On an OLED screen, one sees physical characters or colored pixels—physical elements that literally constitute images. From a speaker, one hears physical sound waves—audible physical phenomena. But information itself? Never. Is this not odd?

One might reply that no one has ever seen even electric charge or atoms. But this is only partially true. First, because they are detectable, as, in principle, all entities in physics should be. For example, atoms, which were once thought to be too small to be seen (even though Newton believed they would have become visible within a few decades during his lifetime), have eventually been visualized [17]. But, obviously, this is only a particular case of a general rule: anything is physically observable and hence measurable if it produces an effect on something else, which in the case of measurement is another object chosen for its reproducibility and commonly called the measuring apparatus. More generally, something is detectable because it produces effects. A rather straightforward example is the scale. When you step on a scale, the needle moves because your body exerts a certain force on the spring inside. From the intensity of this force, the displacement of the scale’s needle is determined—one that has the appropriate dimensions to eventually be seen. This chain of effects, which starts from a physical phenomenon—no matter how esoteric (whether it is the weight of a body or a graviton, a muon, or the viral charge contained in blood) —from physical effect to physical effect ultimately produces a phenomenon that is observable through the senses (sight is usually the most convenient). This chain of causes and effects was explicated by the physicist Ernst Mach in the nineteenth century and later became the core of the measurement problem in contemporary quantum mechanics [18,19,20]. Information is epiphenomenal and thus undetectable and invisible. As I will argue later, fundamental physical entities must satisfy the so-called Eleatic principle—namely, that something must do something (or be causally effective) to qualify as a fundamental physical entity. If this is not the case, the concept will correspond to an epistemic construct, an abstract object, or will just be void.

In the following sections, we will critically examine the foundations of the notion that information is a physical entity, revealing how it has functioned as a disguised form of dualism, an intuitive but misleading metaphor, and an epistemically unnecessary construct. We will begin by tracing how the transition from the immaterial mind to information has shaped contemporary thinking, showing how information has inherited the explanatory role once attributed to immaterial substances. Next, we will deconstruct the pervasive metaphor of information as a flow, demonstrating that it lacks physical grounding and is conceptually misleading. This will lead us to the fallacy of simple location, highlighting how information has been improperly reified as a spatially identifiable entity. Finally, we will reassess Shannon’s formula, revealing its true nature as a probabilistic measure rather than evidence of an ontologically distinct informational substance. Through this analysis, we will argue for a deflationary perspective that dissolves the alleged reality of information and clarifies its proper role as a heuristic tool rather than a fundamental constituent of the physical world.

## 2. Why Do We Think Information Is Real?

Before questioning the existence and nature of information, it is essential to understand why information has come to be regarded as a physical constituent of reality—a relatively recent development. The concept of information was not deemed necessary before the emergence of the so-called information age. In physics, the need to describe reality in informational terms emerged only in the 1950s, influenced by the intersection of quantum mechanics and information theory. This shift encouraged a form of implicit idealism, arguably more scientifically respectable than traditional Cartesian dualism. After all, foundational figures such as Newton, Darwin, Maxwell, and Mach did not require the notion of information to explain the physical world, and one could argue the same applies to Bohr, Schrödinger, and Boltzmann [21,22].

Yet the question remains. If information is taken to be a “bona fide physical entity” [23], does this not lead to some form of dualism that might be avoided by keeping a more deflationary attitude? Do we really need to assume that it is a physical component of reality, as many authors have maintained [2,11,12,24,25]? There is a fundamental dilemma concerning the physical existence of information, one that has surprisingly attracted limited attention: either information exists physically or it does not. If information does exist physically, it cannot merely coincide with the physical processes themselves—otherwise, it would add nothing new. Therefore, it would have to be something distinct from these processes, implying a form of ontological dualism. In other words, physical processes would then exhibit a dual nature: they would be physical events on one side and carriers of informational content on the other.

Overall, if we consider the scientific literature, there are three very strong reasons why scholars and laypeople have begun to consider information as something tangible: the shift from mind–body dualism to information–machine dualism, the metaphor of information as a substance that flows (is created, transferred, carried, stored, etc.), and the fallacy of the simple location. In addition, of course, there is the influence of Shannon’s formula and its intricacies that render it opaque and mysterious for those whose talents lie outside mathematics. The latter issue will be addressed in depth in a separate section.

### 2.1. Dualism in Disguise

As for the transition from the immaterial mind to information, its cultural appeal is quite evident. After all, information is invisible, eternal, can be transferred from one physical medium to another, and resides in the information cloud. If we had asked a theologian of the seventeenth century to indicate some attributes of the mind or the soul, he would have probably agreed. The mind was invisible, eternal, could transfer from one body to another, and resided (presumably) in heaven. The analogies are very strong. Let alone that the mind was a close relative of entelechy and thus of the Platonic form. The transition from Platonic forms to information becomes almost automatic. When Platonic forms were identified with the individual minds of Cartesian dualism, the ground was prepared for the next step [26]. Moreover, the closeness between Platonic forms and mathematical entities creates a smooth conceptual path from modern information—usually expressed in terms of numbers and computations—and thoughts and ideas (which are the modern words for forms).

Actually, since dualism fell into disrepute after the first half of twentieth century, information has, in many ways, taken the place of the immaterial realm. To use the words of Shannon himself, “information theory has […] become something of a scientific bandwagon [that] has perhaps been ballooned to an importance beyond its actual accomplishments. Our fellow scientists in many different fields, attracted by the fanfare and by the new avenues opened to scientific analysis, are using these ideas in their own problems.” [27]. This shift is not coincidental but is deeply rooted in the history of philosophy and the evolution of scientific thought. It is fair to argue that since for many, information plays the role of mediator between the physical and the semantic, just as the immaterial mind did in dualism, it is now used to explain phenomena (e.g., cognition, communication, computation) that previously required a non-material mind. Information has replaced the immaterial mind as the explanatory framework for cognitive, communicative, and computational phenomena. This shift responds to the decline of dualism and the need to integrate mind and meaning into a scientific perspective.

The aforementioned concepts of Tononi’s integrated information and Baars’s central processed information are both suitable examples of such conceptual migration from the immaterial mind to information [28,29,30]. Other examples include Chalmers’ pan-computationalism, Floridi’s information structural realism, and Wheelers’ slogan “It from bit” [11,31,32]. One way or another, these perspectives seem to suggest that reality is fundamentally composed of informational structures rather than material objects, effectively considering information the foundational substance of metaphysics. However, they encounter the same issues faced by the metaphysical views they aim to replace. Generally, if reality comprised information, questions about the necessity of matter and the relationship between information and the physical world would demand an answer.

Speaking about information as if it were physical and yet pretending it to be different from physical processes is paying lip service to physicalism. Yet, one cannot have their cake and eat it too. If information were physical, it would have causal power, but then it ought to be detectable, and thus it would be physical. Moreover, who would need information if it were physical? As an ontological addition, information is relevant only if introduces an additional level to the physical world. In short, if information were identical to its vehicles or some property they have, information would just be such vehicles, and thus there would be no need of it. Unless one is covertly missing some form of idealism or even dualism insofar as it postulates the existence of two levels of reality (things and the information carried by things, as in the expression “the information carried by the photon”), why should we add information to the world? As mentioned above, this is the risk of *information immaterialism*: “Now that we are presented with a fundamental physical theory which turns on the notion of information […], perhaps information itself should be recognized as the fundamental constituent of the world. […] Pieces of information are the new-fangled correlates of sense data; Berkeleyan ideas-in-the-mind in the modern dress of the latest theory” [6].

Indeed, dualism between matter and information is often explicit. For example, George Williams speaks of a “codical domain” coexisting with the material one [33]. Similarly, Maynard Smith and Szathmary speak of “the dual nature of life … metabolic and informational” [34]. Finally, Nobel Laureate Sydney Brenner stated that in biological systems, “in addition to the flows of matter and energy, there is also flow of information” [35].

Famously, Rolf Landauer made the strong claim that information is physical insofar as its deletion has always an irreducible physical cost. According to him, “Information is not a disembodied abstract entity; it is always *tied* to a physical representation” ([12], italics mine). While Landauer rejects the disembodied abstraction and thus rules out Platonism, he stops short of full identity. His position remains somewhat **functional**: information is physical because it always requires energy, space, and time to be instantiated or manipulated. By using the expression “being tied to,” he implicitly suggests that information highlights something different than pure causality. More on Landauer later.

Indeed, another physicist, the aforementioned John Wheeler, has repeatedly and unambiguously acknowledged that the notion of information leads to idealism: “It from bit symbolizes the idea that every item of the physical world has at its core—often a very deep one—an *immaterial source* and explanation. Reality, in essence, emerges from the posing of yes-no questions and the recording of equipment-evoked responses. In summary, all physical entities originate from information theory, and this is a participatory universe” (italics added) [11]. It is well known that there is a current of thought in consciousness studies that has viewed positively some kind of panpsychist stance where the conscious observer has a role in fixing the ontology of the world [36,37,38]. The problem is that such views have never provided a positive account of the conscious observer or explained why should it make reality collapse, let alone that fact that the basic idea of a consciousness-induced wave function collapse has been generally discredited in physics.

Overall, in various fields, the temptation to use the notion of information as a kind of new mental substance, to formulate a contemporary and scientifically acceptable version of dualism or idealism, has made its way and won many supporters. However, these positions have not solved the problem; instead, they have created new ones, starting with the ontology of information that is not explained but merely assumed. Additionally, these positions reproduce many of the issues of classical dualism—starting with the interaction between matter and information—and add nothing to the physical description of reality.

### 2.2. The Metaphor of Flow

A second source of confusion is the pervasive and widespread metaphor of information as a flow. In physics as well as in neuroscience, common expressions such as “information is carried by light” or “information is carried by neurons” are deeply flawed. In fact, if we took such statements at face value, we should expect to have, besides photons and neurotransmitters, something else, namely, information. But do we? Is there anything else? Of course, if asked about their ontological commitments, the authors would vehemently deny that they ever advocated any form of dualism between form and matter, between information and matter. Yet many authors use such a metaphor as if it were a valid one, as Floridi observes: “The information flow—understood as the carriage and transmission of information by some data about a referent, made possible by regularities in a distributed system—has been at the center of philosophical and logical studies for some time.” [32].

The flow metaphor, in which one entity serves as a source of information and another as a receiver, is useful in many contexts. However, this does not necessarily mean that something “flows” between these endpoints. It is an oversimplification to liken information to a liquid or electricity (which, incidentally, is often inaccurately described as something flowing). For Lombardi et al., “Information is a substance; it is a new kind of entity in its own right, something that exists in the world.” Its essential feature consists in its capability of “flowing” through the physical space, that is, of being generated at one point and transmitted to another point; it can also be accumulated, stored and converted from one form to another” [39].

Not all scholars share this view. For one, the aforementioned Timpson states very univocally that while it is tempting to think of information as something that flows around, we must resist since “this picture needs to be handled with extreme caution; for picture it is, rather than statement of fact.” The models we use do not literally tell us “how much of what is produced by a source is diluted, or degraded, or adulterated, or leaks away, or is destroyed—or something of the sort—by the time it reaches the [receiver]” [40].

It is crucial to distinguish between Shannon’s concept of information and the everyday concept. The latter considers information the meaning conveyed by “pieces of information,” whereas the former represents an abstract quantity of the process as a whole. Despite this distinction, both are often mistakenly viewed as though information is inherently co-located with the carriers. In the following, I will not address the everyday concept but will instead focus on the misconceptions that have led many laypeople, scientists, and philosophers to erroneously interpret Shannon’s information as a quantity reflecting occurrences within the channel itself. Although this may appear trivial to mathematicians, as highlighted at the beginning, this misconception is prevalent even among scholars and scientists. Information, in Shannon’s concept, is primarily a characterization of what a source and a receiver can do together, not of a message in motion. The key aspect of this characterization is the compressibility of the source’s output, which is not a property that can travel from one place to another—it makes no sense to say that it “moves” from A to B. Message tokens do not “carry” any quantity of information in the same way that a billiard ball carries its mass. The claim that a message token has the information (or carries it) is simply a way of saying that it originates from a source with certain statistical properties and that such properties are replicated by the receiver. Literally, the message neither carries nor has anything. The content is not a property intrinsic to the token itself; it is neither an observable feature of the token at any given moment nor something inherent in its physical composition. Rather, it is a modal and historical property—it refers to what could be done with the message if a suitable communication set-up were in place, and it depends on the source from which the message originated. Yet the widespread usage of such terminology induces most scholars and laypeople to conceive of information as something that is carried or owned by the message—i.e., by the “piece of information.” This does not happen in sanitized cases such as a transmission, but it is easy to fall into the trap in more complex cases such as the memory inside a computer or the complexity of the Internet or the information cloud. Because information does not figure in the direct, occurrent characterization of physical systems (except for sources) but instead remains an abstract and historical feature, it cannot be said to “flow” in the way that energy does.

In short, information is not a kind of physical stuff that can be transported through pipes or carried along trajectories, nor is it the kind of property that obeys a flow equation. While we often speak metaphorically of the “flow” of information, this must be understood differently. Rather than signifying the movement of a substance, it more aptly refers to changes in the locations where something can be known or reconstructed—where new possibilities for learning or reproducing specific items of information emerge. In this sense, when we discuss information flow, we are not talking about a movement of material but about shifts in accessibility and availability, a point that will be further examined in subsequent discussions.

### 2.3. The Fallacy of Simple Location

Another source of confusion, not exclusive to information theory, is what Alfred Whitehead termed the fallacy of simple location [21]. This common assumption posits that entities—whether physical objects, events, or processes—can be fully described as if they existed at fixed, isolated points in space and time. Any object or event is often considered confined to a specific point or region in space and time, with its properties being regarded as intrinsic and independent of its surroundings [41]. This is a commonsensical view based on our experience with the properties of human-sized objects like apples, chairs, cars, and buildings. It is a common misconception shared by many scientists and laypeople. Yet many physical quantities do not fit into such a simplistic picture. For instance, take potential energy. Where is it? It does not have any location. It is not located where, say, a physical body at a certain location is, since by changing the context and without modifying the body in any way, the potential energy might be totally different. The issue as to whether having a simple location is necessary for being a concrete particular is a complex one that I will not address here. For some authors, actually, lacking the simple location is evidence that something is not a physical concrete entity—it is the so-called non-spatiality criterion [5,42].

Regardless of the solution to this problem, it is evident that very often, both scholars and laypeople have such a model in mind. Consequently, when they encounter a phenomenon, they tend to assume that it must be simply located. As a result, it is commonly held that, since information is physical, it must have a specific location, which is more or less where its carriers are. Hence it must be situated near neurons, characters, neurotransmitters, memory cells, capacitors, wires, or electromagnetic waves. Sadly, there is nothing there besides physical processes and physical stuff with actual causal properties. No information has ever been found anywhere. Only “pieces of information,” which are nothing but “pieces.”

In fact, there is nothing surrounding these physical mediums except the physical mediums themselves, which are tangible objects with defined locations. For instance, a neurotransmitter like a molecule of acetylcholine is clearly located in its synaptic vesicle awaiting release. Similarly, a particular state in an SSD memory capacitor is a significant physical property of a solid-state structure. We can determine where a certain field has reached a new potential, allowing it to be read by an appropriate device and used, for example, to activate LED screens. Once again, is there an additional entity with a simple location near these physical carriers? There is no evidence to support this notion. The message is not simply located where its alleged “carriers” are.

Furthermore, consider how the choice of words—such as referring to “carriers of information,” “pieces of information,” or “vehicles of information”—strongly implies the presence of something being carried. However, this inclination is inherently question inducing. It does not constitute proof that neural activity carries something. Instead, we should scrutinize the prevalent terminology.

In this regard, Timpson notes, “We should be troubled if we were led to think of (quantitative) information as being a kind of (oddly) inscrutable stuff attached to messages” [6] (p. 36). He criticizes the widespread tendency to refer to information as some kind of stuff that “slops about, sometimes managing to transfer in part or whole onto other systems, sometimes not. The mistake here, of course, is to think of quantity of information along the lines that are appropriate for a quantity of a concrete stuff (substance), for example, of milk or sugar: to think of a source of information like the source of a river; to mistake what is an abstract mass noun for a concrete one” [6] (p. 36) Timpson warns against the misconception of treating information as a simply located physical substance like milk or sugar rather than as an abstract concept that is mistakenly perceived as having physical properties.

To summarize, the fallacy of simple location refers to thinking about information in an overly simplistic way. This does not mean that, once avoided, information will necessarily exist. On the contrary, a strong deflationary view is argued for. However, exposing this fallacy is helpful, as it allows for the deconstruction of many motivations underpinning our conception of information. In essence, the “fallacy of simple location” is the mistaken belief that objects or events can be fully understood as if they occupied fixed, independent positions in space and time. Whitehead’s critique emphasizes that reality is fundamentally relational and dynamic, and understanding the interconnections between events is essential for comprehending the true nature of the world. Modeling information as something located near its supposed carrier is another misleading metaphor that should be avoided, and yet this does not mean that information is real.

## 3. Information Revisited

In the previous section, we saw to what extent powerful metaphors can lead many to consider information something physical, almost like a moving fluid or an entity that can be easily located (like electricity or particles). Of course, no one is so naïve, but the scientific and everyday usage of this notion is strongly biased in this sense, and the force of these metaphors in guiding discussion and reasoning should not be underestimated. As mentioned earlier, I now present a series of arguments that weaken the solidity of the notion of information understood as a physical substance. They show that there are strong reasons to doubt of its existence and prepare the ground for the deconstruction of Shannon’s formula.

### 3.1. Measurement and Calculation

As aforementioned, information is undetectable, but not in the sense in which, say, atoms or quarks are—namely, that they cannot be seen by the naked eye. In fact, in the case of atoms or very distant objects, sooner or later, with the right instruments, it will be possible to create a causal chain that will allow for their detection. Information is undetectable in a deeper sense. In fact, information can never be observed in a causal (or physical) sense because—and here, deep doubts should begin to emerge—it is causally ineffective or epiphenomenal. Information is invisible and undetectable, just like centers of mass or calories are. We do not detect calories qua calories, we measure proteins and carbohydrates, fat, sugar, and other molecules. Likewise, we do not detect or measure centers of mass but objects. I will argue that information is undetectable in the same way in which the Pythagorean theorem is undetectable, the way in which abstract concepts are. They are not part of the physical world but rather of the epistemic world. Consider a set of domino tiles that can be raised or lowered. Each tile, under the proper agreement, could indicate a positive or negative response, an, using the standard Shannon paradigm, one could say that it *contains* a certain *amount* of information. Assuming that the positions of the tiles (up, down) are equally probable, one could calculate an amount of information equal to one bit per tile; therefore, for N tiles we have N bits. Do we see/observe/measure the information “contained in” or “carried by” the tiles? Of course not. The quantity of information is not an absolute property of a piece of information because the very same piece of information can be associated with different contexts that will have different Shannon entropies associated with them.

For instance, consider the same physical element—a space–time portion such as my empty desk. Does it contain information? Can we answer this question by measuring it? Clearly not. There is no measuring instrument in the world that can determine whether a given physical system—say, my empty desk—contains information or not. The reason is simple: information is calculated on the basis of the hypotheses we make regarding how that physical system is used and inserted into a certain probabilistic context. Returning to the desk, different contexts would lead to completely different quantifications and therefore attributions of information. For example, I might have agreed with Jane that if I leave something (anything) on the desk, it means I will not come home for dinner. In this case, the absence of objects on the desk would mean that I come home for dinner and thus be worth one bit. But I could have agreed on a different code based on what “might” lie on my desk: nothing means I will not come home for dinner; my “yellow hat” means I will come alone; my “sweatshirt” means I will come with one friend; my “Spider-Man mug” means I will come with many friends. The list could continue indefinitely. In these circumstances, the empty desk (identical to the previous case) would be worth two bits of information. With a much more sophisticated agreement (and in this case, admittedly, a bit maniacal) the same empty desk may be used to store an arbitrary amount of information. The reason is that information is not located where it is normally believed to be (neither on the surface of my desk nor in the capacitors of a RAM chip). Actually, information is not located anywhere. According to Shannon’s definition, information depends on the prior knowledge that one is assumed to have about a physical system—knowledge that concerns mainly the use and the probability of the alternatives of that system. The fact that the same identical system can have a completely different amount of information (essentially from 0 to ∞) should clearly imply that information is not something physical and is not localized in a particular point. However, I will return to this later. To recap, physical quantities are measured thanks to their causal efficacy. By contrast, information is calculated by stipulating a priori knowledge of system statistics and/or the system context.

Information is calculated, not measured. It cannot be measured because it does not have any causal efficacy. This distinction is crucial. Physical quantities are measured—mass by means of a scale. Conversely, information is computed through calculations (such as using Shannon’s formula or Kolmogorov’s complexity) based on hypothetical estimates of system probabilities rather than actual occurrences or existing conditions. Here, as I declared at the outset of this paper, I must stress my deflationary approach to objective probabilities. I am unabashedly skeptical about objective probabilities, like other authors [43,44,45]. For instance, E.T. Jaynes stated that “mathematicians generally […] have a tendency to use language which implies that they are proving properties of the real world […] these theorems are valid properties *of the abstract mathematical model that was defined and analyzed*. […] no mathematical model captures every circumstance that is relevant in the real world. Anyone who believes that he is proving things about the real world, is a victim of the mind projection fallacy“ [43] (p. 75). Probabilities do not exist in the world; there are physical processes that we can count, and by making appropriate assumptions, we can think that they will repeat themselves in a similar way and thus with the same frequency. I can count the number of times a coin toss results in heads and think that if I did this an infinite number of times, I would approach an abstract ideal quantity such as the probability of one-half. But there is no actual probability of one-half in the world that serves as the limit of my sequence of tosses. Limits are a mathematical abstraction, and the notion of objective probability as something in the world that we approximate by means of the limit of a frequency is a myth of statistics textbooks. I can count the number of dinner invitations that were accepted over the course of my life, but there is no probability that determined the outcome. There are only things, and we can count what happens, but probabilities, in themselves, do not exist. Thus, from this perspective, I must confess my deflationary position regarding the existence of objective probabilities, and although I cannot elaborate further here due to obvious space constraints, I emphasize it in order to make the premises of my reasoning clear.

Physical measurements are a totally different ballgame. They result from the causally efficacious interactions between entities—the aforementioned effect of the mass of a body on a spring inside a scale or the change in the physical state of liquid mercury inside a vial due to the proximity of a gas made of very fast particles (summarized by saying the gas has a high temperature). We often resort to intermediate abstract quantities, such as temperature, but we know that such an abstraction is nothing but the average kinetic energy of the particles in a system, which in turn is a measure of the microscopic motions—translations, rotations, and vibrations—of the particles themselves. Thus, such measurements are the outcome of a causal process going from the measured physical property toward the physical measurement apparatus.

A mandatory caveat: nothing I said implies a claim that something is not measured if you have to perform a calculation. Of course, in many practical circumstances, calculations may help to estimate quantities that would otherwise be difficult to ascertain in practice. However, if something is physical, in principle, it is always possible to trace all our numbers to a concrete physical effect of the measured property. This is not the case with information.

Therefore, in physical terms, information cannot be said to exist but rather is an abstract concept. As mentioned above, information is not spatially located, which—as we will see—suggests it is an abstract concept [46]. Where it is supposed to be processed, transmitted, or stored, there is nothing except physical supports that, under appropriate assumptions, can be described using the formalism offered by Shannon’s theory. The crux of the matter—which I will get back to—is that information depends on what one knows about the system. In short, exactly the same physical subset may “carry” different amounts information based on our knowledge about the system.

### 3.2. Eleaticism and Eliminativism

Let us go further. Let us return to the example of domino tiles (or any other system, be it an abacus, a quipu string, RAM, SSD memory, or a sheet of paper). Suppose we have some tiles and can provide a complete causal description of them. It is a deterministic system. The relevant question in this context is as follows: Under these circumstances, besides the tiles, is there something additional that we call information? Or do the tiles exhaust the ontological requirements of the system?

If I have a complete description of the tiles, I am able to fully describe and predict their future evolution. Even if there are stochastic components—say, a quantum tile—I will account for them and, although I will not predict specific events, I will still describe its future evolution (like predicting the average number of heads and tails for a reasonably large number of coin tosses). The point here is not, of course, prediction per se but the more fundamental issue that once one has a complete physical description of a system, there is no space left for information. If I knew the exact position of a coin, probability would disappear; likewise, if all the properties of a physical system were fully given, information would become useless—just like the notion of the center of mass. As E. T. Jaynes stated, mathematicians “invent the dignified-sounding word randomization […] This term is, evidently, a euphemism, whose real meaning is: deliberately throwing away relevant information when it becomes too complicated for us to handle […] an antidote is needed for the impression created by some writers on probability theory, who attach a kind of mystical significance to it” [43] (p. 73). Probability is a measure of our ignorance, and information is a measure of our surprise, the four notions being intimately intertwined.

Therefore, from a causal point of view, do we need information as an additional component of reality? Intuitively, the answer is negative. The world is already causally complete without information, which adds nothing causally relevant. The description of causally relevant factors plus any stochastic factors exhausts the description of the world. The burden of proof is usually on the shoulders of those who defend a positive claim—the causal relevance of an abstract object like information, in this case [32].

Suppose information were causally relevant and, therefore, modified the course of events. We would have to admit that, in that case, besides the basic events, there is at least one more fact or event that modifies the course of events, compared to a scenario in which only the basic events occurred. This is evidently contradictory. It is absolutely not true that, as Luciano Floridi suggests, in addition to physical states, there exists something called information that plays a role in determining the evolution of a system [32]. If there were, it would be physical, and thus it would not be information but just another physical fact available to be measured and observed. Everywhere, *events happen, blissfully unaware of the fact that someone describes them in informational terms*. The physical world does not need information. Physics, as a mathematical description of the world, might need information. Yet this is a different matter. Therefore, there is no additional fact called information. And there cannot be because, if there were, we would fall into the case of causal redundancy, that is, having two causes that produce the same event when one would suffice.

The question of existence and causal efficacy arises: if something has no effect, it does not exist. Here, I assume that existence and causal efficacy are coextensive, as stated, in slightly different ways, by the principle of causal inefficacy, the Eleatic principle or Eleaticism, and Alexander’s dictum [5,46,47,48]. While there might be slight differences, I will adopt Cowling’s formulation of Eleaticism: “Necessarily, some entity x exists if and only if x is causally active” [46]. While this formulation means that the absence of causally inactive entities in the actual world is necessary but not sufficient for Eleaticism to be true, I will be satisfied with a nomological version of it. Namely, it is enough that in this universe, what exists is also causally active and vice versa. There is no pretense for metaphysical necessity. As a substantive ontological claim, Eleaticism requires a robust notion of causal activity to avoid triviality. It rejects interpretations that equate mere existence with causal activity, even though such views would technically support it. At this stage, *being causally active* is assumed to be a broad condition that different entities fulfill in different ways, and I will proceed under the assumption that both objects and properties are causally active.

In general, Eleaticism rules out of existence all supervenient properties simply because they are epiphenomenal and thus have no causal role in shaping reality. A very strong defendant of this view is the philosopher Jaegwon Kim, according to whom, “there may really be not much difference between eliminativism and epiphenomenalism. For a plausible criterion for distinguishing what is real from what is not real is the possession of causal power.” [47] (p. 119). Other authors have argued that existence and causal efficacy are pretty much the same. For instance, Trenton Merrick argued that “to be is to have causal powers” [49] (p. 81). Likewise, many authors have supported eliminativism as a form of neat ontological stance [47,49,50,51,52,53].

It is tempting to consider entities that have no obvious causal relevance for reality—but why should we? Do they truly add anything to the world we live in? For instance, one might argue that structures such as spacetime are legitimate physical entities. Yet spacetime can either be understood, as in the Kantian tradition, as a transcendental condition for causally relevant entities or—according to certain quantum mechanical interpretations—as an actual ingredient of reality, endowed with causal efficacy. In the former case, it would not exist; in the latter case, it would exist, and the Eleatic principle would apply. However, I must admit that a thorough discussion of Eleaticism lies beyond the scope of this paper. I will therefore limit myself to stating as a fundamental premise of this work the view that an entity must be causally active in order to be taken empirically seriously.

Without further metaphysical nuances, I stress that Eleaticism is one of the methodological cornerstones of science, where entities and properties exist because they produce effects. In this sense, epiphenomenalism usually leads to the elimination of something from the list of existing entities. If something is epiphenomenal, the world does not need it. Neither does science. In the case of information, we have seen cases and arguments that should undermine our confidence about its causal efficacy—information per se has no causal efficacy; rather, the underlying processes that we describe through information have it.

### 3.3. Causal Redundancy

Causal redundancy occurs when two distinct entities are claimed to perform the same causal role or bring about the same effect independently. In such cases, one of the entities is often considered superfluous, as its contribution to the causal outcome is either unnecessary or duplicative, raising questions about explanatory economy and ontological commitment. Which one has to be set aside? While this question cannot be addressed fully here, a general criterion might be to choose that for which its existence leads to a more parsimonious ontology.

Consider a chocolate bar and read from the label the amount of various components: these will be divided into a certain amount of proteins, fats, saturated and unsaturated lipids, fibers, and sugars. These components, if weighed, exhaust the weight of the food in question. Say we have a tablet to chocolate with a label that reads (for 100 g): 55 g of lipids, 14 g of carbohydrates, 10 g of protein, 1 g of salt, 15 g of fiber, and 5 g of water. If we asked a chemist to extract the above components one by one (proteins, fats, lipids, etc.), once all were removed, nothing would be left. And yet the label reads that the food also contains a certain amount of calories (specifically 592 Kcal). The whereabouts of these calories can be clarified by understanding that they are not a material component of food but rather a measure of what the food can do in the right circumstances. Specifically, one calory indicates the capacity to raise the temperature of one kilogram of water by one degree Celsius through combustion in the presence of oxygen or during metabolic processes like the Krebs cycle. For instance, on the moon, where there is no oxygen, food would not generate any calories, as combustion cannot occur. Thus, calories are analogous to information in that they are not intrinsic physical entities within matter, organisms, or food but symbolize the potential actions that the food can perform under suitable conditions. In a sense, calories are not literally there; they are an abstract way to express what we know food would do if it were placed in the appropriate circumstances.

Information is in many ways analogous to calories, which at least represent, under appropriate conditions, the energy and work a physical system can perform. Significantly, work is related to entropy and thus to the order of a system. However, reifying order as something that exists beyond the elements arranged orderly is not only unnecessary but ontologically redundant. If, besides the elements composing a system, order (even understood as structure) existed too, causal redundancy would occur. By the same token, information does not exist as a substance, material, or principle. It is not an additional fact. Information is an abstract way to quantify, given a certain situation assumed to be known a priori, what the physical system could do concerning an abstract, but not physical, space of possibilities. My contention is that *information is an abstract concept, that is, a concept that is epistemically useful but does not correspond to anything in the real world*.

One last useful example is offered by the concept of the center of mass: given a complex system, the center of mass is the point in space where, from the perspective of another body, it would be equivalent if all the mass were concentrated there. For example, Earth’s center of mass is approximately at its geometric center. We all feel attracted to the center of the Earth as if at that point there were a very powerful attractive principle pulling us toward it.

Obviously, at the location indicated by the center of mass, there is nothing, and the gravitational attraction toward that point is simply the vector sum of the attraction vectors of each particle that composes our planet. Given the symmetry in the mass distribution of our planet, their composition points quite precisely toward the planet’s geometric center. The simplification offered by the center of mass is so effective that it is not uncommon to hear statements like “every object is attracted to the center of the Earth.” In bygone ages, the standard explanation was that we were pulled *by* the center of the Earth. Today, every schoolchild knows that nothing attracts us to the center of the Earth. Moreover, the very idea that there must be something in the direction of the force that is pulling is a naïve inference based on analogies with ropes and arms. Gravity is a case of a central force. Central forces are a special case where the force is always directed toward or away from a fixed point. Many real-world forces—particularly those involving velocity dependence, rotation, friction, and complex field interactions like magnetism—do not satisfy this condition.

Here, I would like to draw a further distinction among abstract objects. Some of them are innocuous, since they are straightforwardly outside the physical world. For instance, the Pythagorean theorem is obviously abstract; nobody expects to find it anywhere. The center of mass, on the other hand, is different, and it might lead many to believe that it is a real physical entity, which it is not. I would like to call the latter kind of abstract objects pseudo physical concepts to stress that they might mislead people into assuming they are physical entities. In the past, there have been many cases of such pseudo concepts that were later rejected—Ptolemaic epicycles, calories, phlogiston, luminiferous ether, and, who knows, possibly dark matter if MOND theories have any merit, are all suitable examples [54,55].

To recap, notions such the center of mass or calories are mathematical abstractions with no direct physical counterpart. By the same token, information too is an abstract object, useful for describing reality but non-existent outside our descriptions of the world. Calculated but not measured. Furthermore, the causal closure of the physical world implies that there is no room—under the penalty of causal redundancy or, worse, of causal inefficacy—for the existence of an additional cause or an additional entity beyond physical entities. Abstract objects are doomed to be epiphenomenal and thus to not exist. If information is epiphenomenal, as it seems to be, information will not exist [6,23].

## 4. Shannon Revisited

In the previous sections, I argued that the concept of information—introduced only in the 1950s—gained widespread recognition not only due to its undeniable technical merits but also because it reinforced an intuitive view of information as a quantifiable and transferable entity. I have presented a series of arguments revealing the cultural and metaphysical factors that have contributed to the widespread belief in the existence of information as some kind of stuff. However, even after considering these arguments, many readers may still resist embracing a deflationary view of information, largely due to the influence of Shannon’s formula. This landmark theory, which has dominated introductory courses in information theory, communication, and artificial intelligence, has shaped how information is perceived. It is unlikely that this confidence will waver unless a radical reassessment is undertaken. Shannon’s formula, however, can indeed be mathematically revisited, showing that—despite its undeniable utility—it does not introduce a new ontological entity called information. Rather, it provides a convenient logarithmic and additive formalization of the probabilities.

Without further ado, let us start from Shannon’s classical formula as he presented it in his original paper and as it is nowadays presented ubiquitously [4]:

(1 or H) $$H=-\sum_{i=1}^{N}p_{i}Log(p_{i})$$

While this equation is very well known, its meaning is rather obscure. Even the textbooks rarely justify it. It is almost always presented as a starting point. For example, Christopher Bishop, in an academic textbook about machine learning and statistics, introduces H saying that it has been chosen because of “a rather heuristic motivation [and because] it indeed possesses useful properties” [56].

Most students (and scholars too) read and utilize this under the impression that H should represent a quantity of something that exists physically and has real causal powers. Yet, as we have seen in the previous paragraphs, there are many good reasons to deny this. In this section, I will also put forward good mathematical reasons.

Equation (1) (H), best known as the Shannon entropy equation, does not offer an obvious relationship with the physical world (as might be the case with other equations, for example *F* = *ma*). The summation of the product of probabilities by their logarithm is epistemically opaque. Shannon himself justified the formula in pragmatic rather than theoretical terms [4,57]. In fact, Shannon started with Hartley’s original formula [58], which was introduced as “a derived measure which does meet the *practical* requirements,” mostly because “the logarithmic measure is […] *practically* more useful” ([4], italics mine). Whereas Hartley “*arbitrarily* put the amount of information proportional to the number of selections and [chosen] the factor of proportionality as to make equal amounts of information correspond to equal numbers of possible sequences”(p. 19), Shannon was looking for a handy way to express the probability of the occurrence of each long sequence made of symbols, the occurrence of which is assumed to be mutually independent. He fine-tuned his position and stated that “H is approximately the logarithm of the reciprocal probability of a typical long sequence divided by the number of symbols in the sequence” [57]. After that, he also considered cases of mutual dependence of symbols with Markov chains. He was not looking for another state of matter but for a working solution to a practical problem.

Shannon was very explicit about the (non) physical nature of H. He was tempted by logarithmic functions because, as an engineer, he noticed that parameters of engineering importance—such as time, bandwidth, number of relays—often tend to vary linearly with the logarithm of the number of possibilities. For example, adding one relay to a group doubles the number of possible states of the relays. It adds 1 to the base 2 logarithm of this number. Doubling the time roughly squares the number of possible messages or doubles the logarithm, etc. More importantly even, he wanted something close to an intuitive feeling about physical measurements. He wanted a formula showing an additive and linear behavior. For instance, two punched cards should have twice the capacity of one for information storage, and two identical channels twice the capacity of one for transmitting information. Logarithm qualified. Finally, Shannon wanted something mathematically manageable. Many of the limiting operations are simple in terms of the logarithm but would require clumsy restatement in terms of probabilities. So, Shannon introduced H, not because of any deep physical meaning but rather because it is “practically more useful,” “mathematically more suitable,” and “nearer to our intuitive feelings.” Putting it all together, he set three mathematical heuristic constraints (pp. 18–20 and 82–83):

1. H should be continuous.
2. If pi=1N, then H must be a monotonic increasing function of N.
3. If a sequence of symbols is broken down into two or more subsequences, the H of the original sequence should be the weighted sum of the Hs of the subsequences.

Eventually, Shannon provides a mathematical proof that the only working solution is Equation (1). However, “the real justification of these definitions […] *will reside in their implications*” (p. 19, italics mine). In other words, the formula is a mathematical tool expressing the probability of a sequence of independent events (e.g., the symbols transmitted in a message) in an appropriate and manageable way, offering a linear additive dependence with subsequences.

### 4.1. From Information to Probabilities

To demonstrate that information is merely probabilities, we need to examine Shannon’s formula more deeply. The point here is to illustrate step by step the mathematical derivation from Shannon’s formula to independent probability. Let us start from Equation (1) (H onwards) once again:

(H) $$H=-\sum_{i=1}^{N}p_{i}Log(p_{i})$$

We can move the minus sign on the left and rewrite both terms as exponents:

(2) $$2^{-H}=2^{\sum_{i=1}^{N}p_{i}Log(p_{i})}$$

We can focus on the right side and proceed with standard first-grade derivations:

$$2^{\sum_{i=1}^{N}p_{i}Log(p_{i})}=\;=2^{p_{1}Log\;p_{1}+p_{2}Log\;p_{2}+\ldots {+p}_{N}Log\;p_{N}}=\;=2^{p_{i}Log\;p_{1}}\cdot 2^{p_{2}Log\;p_{2}}\cdot \ldots \cdot 2^{p_{N}Log\;p_{N}}=\;=2^{Log\;p_{1}\cdot p_{1}}\cdot 2^{p_{2}Log\;p_{2}}\cdot \ldots \cdot 2^{p_{2}Log\;p_{2}}=\;={\left(2^{Log\;p_{1}}\right)}^{p_{1}}\cdot {\left(2^{Log\;p_{2}}\right)}^{p_{2}}\cdot \ldots \cdot {\left(2^{Log\;p_{N}}\right)}^{p_{N}}=\;={\left(p_{1}\right)}^{p_{1}}\cdot {\left(p_{2}\right)}^{p_{2}}\cdot \ldots \cdot {\left(p_{N}\right)}^{p_{N}}=$$

(3) $$=\prod_{i=1}^{N}{p_{i}}^{p_{i}}$$

This derivation begins to look very close to the formula for independent probabilities:

(4) $$P\left(AB\right)=P\left(A\right)P(B)=\prod_{i=1}^{N}p_{i}$$

However, this is not good enough. Luckily, we can reorganize Equation (3) so as to remove the exponents in the following way. A numerical example will help. Consider having any number expressed as a potence. For instance, $4^{5}=4^{2}\cdot 4^{3}$. In general, any $x^{y}=x^{a}\cdot x^{b}$ as long as y=a+b. We can then generalize and say that any $p^{p}=\prod_{j=1}^{L}p^{d}$ as long as p=L⋅d. This is just high school calculus. In this way, though, we can break down any factor ${p_{i}}^{p_{i}}$ into a product of many smaller factors ${p_{i}}^{p_{i}}=\prod_{j=1}^{L}{p_{i}}^{d}$. Is there a suitable d that will hold for all factors ${p_{i}}^{p_{i}}$ in Equation (3)? Yes, there is. Let d∈Q be the greatest rational common divisor of all $p_{i}$ (assuming $p_{i}\in Q,\forall i$); d is the greatest rational such that $q=\frac{p_{i}}{d}\in N,\forall i$, which is a formal way to state that d is the greatest common divisor of all $p_{i}$. Is the limitation to the domain of rational numbers a severe reduction in the generality of this reduction? I doubt this for two reasons. First, although real numbers are foundational in the mathematical formulation of physical theories, their necessity in describing physical reality is questionable. All empirical measurements are finite and limited by instrumental precision and fundamental physical bounds [59,60]. The Bekenstein bound further suggests that any finite region of space can be dealt with only a finite measure of information. Consequently, as a first attempt, I believe that rational numbers may offer a sufficiently adequate domain. More generally,

(5) $$d={gcd\left(p_{i}\right)}_{i=1}^{N}=\frac{{gcd\left(a_{i}\right)}_{i=1}^{N}}{{lcm\left(b_{i}\right)}_{i=1}^{N}}$$

where *gcd*() is the greatest common divisor, *lcm*() is the least common multiple, and $p_{i}=a_{i}/b_{i}$, $a_{i}\in N,b_{i}\in N,\forall i$. By means of d, Equation (3) can then be rewritten as

(6) $$\prod_{i}^{N}{p_{i}}^{p_{i}}={p_{1}}^{p_{1}}{\cdot p_{2}}^{p_{2}}\cdot \ldots \cdot {p_{N}}^{p_{N}}={{\tilde{p}}_{1}}^{d}{\cdot {\tilde{p}}_{2}}^{d}\cdot \ldots \cdot {{\tilde{p}}_{M}}^{d}$$

whereas each factor ${p_{i}}^{p_{i}}$ is rewritten as a product $\prod_{k}^{K_{i}}{\left(p_{i}\right)}^{d}$ where $K_{i}=p_{i}/d,\;K_{i}\geq 1.$ Of course, if $d=p_{i}$, $K_{i}=1$. Conceptually, each probability $p_{i}$ corresponds to an event $x_{i}$. By performing the substitution in Equation (5), the same event is now a set of $K_{i}$ events ${\tilde{x}}_{i}$ with probability ${\tilde{p}}_{j}$ which is, in turn, equal to $p_{i}$ for each subset i. Thus, each $p_{i}$ can be revisited by—and thus substituted with—a series of independent events ${\tilde{x}}_{i},\;1\leq i\leq M$, each having the probability ${\tilde{p}}_{i}$. I will provide below a numerical example to make this substitution straightforward.

While this method may look a bit complicated, it allows us to rewrite Equation (3) as

(7) $$\prod_{i}^{N}{p_{i}}^{p_{i}}=\prod_{j}^{M}{{\tilde{p}}_{i}}^{d}$$

Since all factors have the same exponent, it is finally possible to move it outside of the product operator and obtain

(8) $$\prod_{j}^{M}{{\tilde{p}}_{i}}^{d}={\left[\prod_{j}^{M}{\tilde{p}}_{i}\right]}^{d}$$

This rewriting allows us to go back to Equation (2) and rewrite it as

(9) $$2^{-H}={\left[\prod_{j}^{M}{\tilde{p}}_{i}\right]}^{d}$$

Hence,

(10) $$H=-d\cdot Log\left(\prod_{j}^{M}{\tilde{p}}_{i}\right)$$

Since d=1/M, also (finally),

(11 or HR) $$H=-\frac{1}{M}\cdot Log\left(\prod_{j}^{M}{\tilde{p}}_{i}\right)$$

We can easily verify whether Equation (11) (HR onward) is equivalent to Equation (1) (H), and it is. For instance, if the elements are equiprobable symbols, $p_{i}=\frac{1}{N},\;M=N,{\tilde{p}}_{i}=p_{i}$, Equation (11) would lead to

(12) $$H=-\frac{1}{N}\cdot Log\left(\prod_{i}^{N}p_{i}\right)=-\frac{1}{N}\cdot Log\left(N^{-N}\right)=-\frac{1}{N}\cdot \left(-N\right)LogN=LogN$$

which is, once again, Hartley’s familiar equation [58]. We can also consider numerical examples to verify whether HR is right. Assume three outcomes $x_{1},\;x_{2},\;x_{3}$, with probabilities $\left\{\frac{1}{6},\frac{1}{3},\frac{1}{2}\right\}$, *N* = 3. The greatest common rational factor is *d* = ⅙. By multiplying all elements according to the ratio $K_{i}=p_{i}/d$, a set $\left\{{\tilde{x}}_{j}\right\},\;M=6$, where each element has a probability $d=\frac{1}{6}=\frac{1}{M}$, we obtain $\left\{p_{1},\;p_{2},p_{3}\right\}\Rightarrow \left\{{\tilde{p}}_{1},\;{\tilde{p}}_{2},\;{{\tilde{p}}_{3},\;{\tilde{p}}_{4},\;\tilde{p}}_{5},{\tilde{p}}_{6}\right\}$. Performing the calculations, H results in $H=-\left(\frac{1}{6}{Log}_{2}\frac{1}{6}+\frac{1}{3}{Log}_{2}\frac{1}{3}+\frac{1}{2}{Log}_{2}\frac{1}{2}\right)≅1.459147917\ldots$; HR results in $H=-\frac{1}{6}{Log}_{2}\left(\frac{1}{6}\cdot \frac{1}{3}\cdot \frac{1}{3}\cdot \frac{1}{2}\cdot \frac{1}{2}\cdot \frac{1}{2}\right)≅1.459147917\ldots$ Success!

In the binary case, luckily, things gets much simpler. Given a series of L binary digits, where H would result in L bits, HR will produce the same value, of course, since $M=2^{L}$, and ${\tilde{p}}_{i}=1/2^{L}$. So, using H, we will obtain

(13) $$H=-\sum^{2^{L}}\frac{1}{2^{L}}Log\left(\frac{1}{2^{L}}\right)=\frac{2^{L}}{2^{L}}Log\left(2^{L}\right)=L$$

And by the same token, using HR, we will obtain

(14) $$H=-\frac{1}{2^{L}}\cdot Log\left(\prod^{2^{L}}\frac{1}{2^{L}}\right)=\frac{2^{L}}{2^{L}}\cdot Log\left(2^{L}\right)=L$$

All is well and good, and this tells us that, given L binary digits, we will have $2^{L}$ possible configurations (or messages), and if there are no co-dependencies, each configuration will have a probability of ${\tilde{p}}_{i}=1/2^{L}$. It is therefore easy to see that ${\tilde{p}}_{i}$ indeed has a physical meaning, which is the probability of each possible configuration. As for the meaning of $\prod_{j}^{M}{\tilde{p}}_{i}$, while I am not prepared to answer this fully at the moment, a direction for further enquiry can be suggested. $\prod_{j}^{M}{\tilde{p}}_{i}$ is the probability inside a space of as many configurations as the possible sequences; thus, it might be seen as the probability of a sequence of sequences. In other words, the product offers the probability of a very specific, fully determined path through the space of all possible sequences. In any case, H is a monadic biunivocal function of $\prod_{j}^{M}{\tilde{p}}_{i}$ and thus, based on dimensional analysis, should have the same physical dimensions of its only argument, which is non-dimensional like all probabilities. H and HR are numerically equivalent. They can be derived from each other. The derivation from Equation (1) to Equation (11) (from H to HR) is complete, and it shows that, dimensionally speaking, information is nothing but probability.

### 4.2. Information and Probability

Therefore, Shannon introduced a formula (H) to convert probabilities into a quantity (information) that allowed him to deal with multiplications and fractions in terms of a linear sum of additive quantities. Does H reveal a new level of reality (the informational one), or is it just a more convenient way to deal with probabilities? In the previous section, Shannon’s standard equation or H was written in the form of HR:

(H) $$H=-\sum_{i=1}^{N}p_{i}Log(p_{i})$$

(HR) $$\;H=-\frac{1}{M}\cdot Log\left(\prod_{j}^{M}{\tilde{p}}_{i}\right)$$

The success of H is due to a series of handy properties (monotonicity, additivity), but its epistemically opaque structure has given many the erroneous impression that it measures some physical quantity. However, HR, which is mathematically equivalent, is more transparent—it does not suggest the existence of another ontological level (the informational one). HR is basically a nonlinear function applied to the probability of independent events, namely symbols, sequences of symbols, and states of a system. HR is a mathematically equivalent version of H that reveals that information is nothing but probability in disguise. Thus, what we call information is actually the probability among the N independent outcomes (or superset M) of possible states in a sequence of symbols in a message or any other system. HR shows how the standard Shannon formula H uses logarithms to turn a product into a sum. While logarithms simplify calculations, they lack physical meaning. Adding logarithms is easier than multiplying fractions, but one must avoid treating these values as if they measured some additional substance. They represent probabilities of independent events, not an additional entity like information.

At this point, it is appropriate for me to explicitly state my deflationary position regarding probability as well and, in this respect, my alignment with those authors—such as E.T. Jaynes and many others [36,37,43,44,45,61,62]—who have challenged the ontic status of probability. However, it is important to note that this additional step toward a deflationary ontology is not essential to the purposes of the present article, which is limited to observing the lack of necessity in attributing to information a further level of existence beyond that of probability. Nevertheless, the issue of whether probability possesses an objective status is not critical to the central claim advanced here, namely, that information can be fully reduced to probability. To recap, it is my contention that HR is more transparent regarding the physical meaning of the underlying phenomena since it shows explicitly the product of independent probabilities. In it, there is no additional entity or quantity. Probabilities exist on both sides of the equation.

Shannon’s formula, mathematically sound and useful, encourages us to think of information as it were an additive substance. The equation itself is harmless, but its mathematical opacity can be misleading. The derivation offered by HR shows that information is nothing but a probability of sequences. To conclude otherwise would be ontologically misleading. The additive structure of H leans toward erroneous ontological assumptions. The characteristics of H—additivity, positivity, monotonicity—have led many to mistakenly perceive H as a substance, given these are typical properties of substances. Yet information realists should provide additional arguments to support their claim.

It must be reckoned, of course, that Shannon’s formula is “mathematically more suitable.” For example, if one used HR to express a mundane fact such as the capacity of a memory card of 1 gigabyte, it would necessitate referring to a system where each state has a probability of 1.1641532e−10, which is highly impractical. Thanks to H, utilizing logarithms and a convenient negative sign provides an additive quantity that simplifies to the familiar figure of 1 gigabyte.

Shannon himself could not help but give the impression that the formula expresses a physical magnitude. The introduction of the term *bit* suggests the existence of something substantial in the physical world. Shannon writes that the choice of a logarithmic base corresponds to the choice of a unit for measuring information, thereby implying that information exists and that his formula measures it. However, this is not the case. The *bit* is an invention, not a measure of some stuff. As historian James Gleick recalls, “One day in the summer of 1949, Shannon took a pencil and a piece of notebook paper, drew a line from top to bottom […] He labeled this axis *bits storage capacity*.” He began listing some items that might be said to *store* information” [63]. This moment was likely a critical step in the development of the concept of information as an entity that can be stored and transmitted. Essentially, the reification of information was historically induced by the practically legitimate choice to use logarithms to represent a product of independent probabilities. Shannon’s formula encourages us to think of information as stuff made of *bits*, whereas there are merely *probabilities*, expressed by fractions. HR suggests that Shannon’s formula is a convenient mathematical transformation with no deep ontological significance.

The widespread notion that information is an additive quantity that can be measured in units such as bits has led many scholars and students to perceive bits as fundamental substances constituting reality. This perspective is appealing because it aligns with the common understanding that the essential components of reality are entities that can be quantified and combined, similar to bricks, atoms, meters, kilograms, and coulombs. Consequently, the additive nature of Shannon’s formula has endorsed the view that bits represent a fundamental building block of reality (see Wheeler). The conceptual misunderstanding has not been resolved over time. On the contrary, it has become more and more entrenched in the literature on information.

It is a fact that if one puts one bit together with another bit, two bits will result, thereby suggesting that they behave like a countable stuff. In contrast, HR shows that what one is really doing is multiplying probabilities. For example, if you have a message of two bits, each having a probability of one-half, each possible sequence in the message will have a probability of one-quarter. If we had used HR to model the probability of such messages, the impression that there is an additive object (two bits) would have disappeared. It would have been clear that we deal with probabilities (which are not substances in themselves) and their products. Of course, 1 + 1 = 2 is just a way of expressing (−Log½)(−Log½) = −Log(¼).

HR allows us to see that the informational level can be removed from our description of reality, and everything will continue to behave the same way. H has no actual causal power—nothing happens because of H. H quantifies in different ways the degree of independent or conditional probability between occurrences at the opposite ends of a channel. The causal power lies elsewhere, prior to the quantities in mathematics. As centers of mass or calories have no actual causal power that is drained by actual physical entities (particles of mass or proteins and sugars), information in itself is only a construct or a tool that summarizes certain causal patterns and matches them with certain logical constructs that humans have learned to use efficiently. Moreover, information has no fundamental ontological status since it does not refer to anything intrinsic to physical systems. If we had used HR, we would have had no need to add the notion of information to describe the probability to exchange messages. Information is thus, as Aaron Levy has argued, a fictional representation, and “informational notions […] are fictional—metaphors rather than descriptions“ [16]. I claim that Shannon and subsequent extensions of information theory have led scientists to place an undeserved ontological status on a metaphorical attitude.

If information is a mathematical construct, as HR shows, then it is untrue that transistors or neurons carry or store information. In computer science, many authors are often encouraged to think that Shannon made a fundamental ontological discovery, namely that flip-flops and transistors contain a substance, information, measured in bits. In textbooks, the concept of information is justified on “heuristic grounds” that are supposed to find justification in its practical utility. Shannon said that. There can be no doubt that the whole field of information theory has been successful. But it has not shown that there is anything other than the physical world. Information theory is not like physics; it is more like geometry. I am neutral about whether abstract concepts are real or not. Surely they are not concrete and causally efficacious physical entities like mass and charge. But information remains an epistemic construct.

To recap, information is not causally efficacious; it is epiphenomenal. Since causal efficacy is a mandatory feature of the physical, this is strong argument to deny that information is physical. Epistemic constructs are not physical. This is a conclusion stemming from the aforementioned application of the Eleatic principle and causal redundancy to physics.

The conclusion is unwelcomed by those scholars who have invested in the existence of a new level of reality as a solution to many problems in neuroscience, psychology, semantics, and philosophy of mind such as intentionality, consciousness, and mental causation (to mention just a few). Not only is information causally ineffective and therefore ontologically non-existent (according to the Eleatic principle), it is also epistemically unnecessary because it is mathematically redundant.

### 4.3. Information Is Not Physical

Is the contention that information is nothing but an epistemic construct incompatible with certain popular claims supporting the notion that information is physical? I am of course referring to the famous position defended by the aforementioned Landauer [12,13,14]. According to him, information is rooted in his work on the thermodynamics of computation. His argument is primarily based on the eponymous principle, which states that erasing information in a computational system has an unavoidable thermodynamic cost, specifically a minimum energy dissipation of $k_{b}Tln2$ per bit erased, where $k_{b}$ is Boltzmann’s constant and T is the temperature of the system [12]. Rightly so, he points out that—whether in a computer memory, a brain, or any other system—*information must be physically instantiated in some form*—magnetization states, electric charges, neuron configurations, etc. Having assumed that, it follows that erasing information is fundamentally tied to entropy increase. For instance, resetting a bit to a standard state irreversibly reduces phase-space volume, leading to an entropy increase in the environment (heat dissipation). In Landauer’s interpretation, this establishes a direct link between information and thermodynamics.

However, Landauer’s argument is sound only insofar as one accepts his premise that information is physically instantiated, which is precisely what he aims to demonstrate. In fact, his conclusion does not necessarily follow. What he actually demonstrated is that when we describe a system using informational terms, certain physical constraints apply—but this does not establish that information itself is a physical entity. What Landauer really showed is that a system described using information-theoretic terms has thermodynamic constraints. However, just because a particular description has physical consequences does not mean that the thing described (information) is an independent physical entity. Landauer’s empirical observation only shows that energy is required to erase the physical structure *described as instantiating information. Information qua information has no role.* In fact, Landauer’s argument is akin to saying that because using a mathematical model of a bridge requires real-world materials (e.g., paper, ink, or a computer), mathematics must be physical. Or, alternatively, we can describe ocean waves in terms of Fourier transforms, but that does not mean that Fourier transforms have an independent physical existence.

If Landauer had merely claimed that information-processing operations, as performed in physical devices, obey physical laws, his conclusion would be uncontroversial. However, his claim that information itself is physical reifies an abstraction: information is not something that exists apart from physical states—it is a way of describing those states. His argument does not rule out the possibility that information is just a useful fiction, much like center-of-mass calculations or probability distributions.

Addressing causal redundancy, we can explore Landauer’s principle’s ontological implications separately from its computational and thermodynamic aspects. While entropy and Shannon’s information share equation forms, they are distinct concepts, making it unwise to draw ontological conclusions solely from their syntax. Landauer’s principle asserts the thermodynamic cost of erasing one bit of information, which merges ontological interpretation with physical fact. Assuming a device contains “one bit of information” is problematic if using the principle to prove physical information’s existence. If information is subjective, the principle pertains more to specific operations than to bit erasure.

Landauer’s principle defines the minimum energy required for computations, but does it mean that “information” is physical? I argue it does not due to causal redundancy. In Landauer’s erasure process, information as a physical entity plays no causal role. The processes are causally closed and can be described probabilistically by showing that system configurations are reduced by irreversible causal actions. This is quite manifest by Landauer’s terminology in statements like “the information-bearing degrees of freedom” [64], “information processing is accompanied by a certain minimum amount of energy dissipation” [64], “information is tied to physical degrees of freedom through a charge, a spin, a hole in a punched card” [12], and “the ultimate physical limits of the information handling process” [12]. Information is never the causal entity doing the actual causal job but something that is tied to the actual causal and physical process. Landauer’s principle is very interesting, but it does not addresses the issue of causal overdetermination, and therefore, it does not provide a compelling argument for the physical existence of information.

In sum, Landauer’s principle is a valuable insight into the thermodynamics of computation, but it does not substantiate the claim that information itself is physical. His reasoning presupposes that information must be instantiated in physical states, yet this assumption does not justify reifying information as an independent entity. Instead, his findings merely show that when we describe a system using information-theoretic terms, the physical processes involved obey thermodynamic constraints. This does not imply that information has a distinct ontological status—rather, it underscores its role as a conceptual tool for describing physical transformations. Given the issue of causal redundancy, Landauer’s principle does not provide a sufficient basis for asserting that information plays a fundamental causal role in physical reality. At best, it reinforces the idea that information is a dependent descriptor, not an ontologically autonomous substance. Thus, while information-based descriptions remain powerful and practically useful, their utility should not be mistaken for ontological necessity.

## 5. Information Reloaded

The importance attributed to information in describing various phenomena has primarily been justified by the prominence of information in computer science practice and, consequently, in the computational modeling of reality. However, from an ontological perspective, there is a notable gap. The existence of information as something additional to the physical world would imply a kind of material–informational dualism, which appears implausible. In this regard, Levy asserts, “However, if one accepts that informational things exist—as, in some form or other, it appears a literal reading of information implies—then it is difficult to say why. The fiction-based view explains what is wrong with a metaphysics of information and obviates the inference from the theoretical role of informational descriptions to the existence of informational things. Informational notions have theoretical significance, but this should not lead us to reify them” [16].

The reification of information exemplifies Whitehead’s fallacy of misplaced concreteness, which occurs when an abstraction (an abstract belief or hypothetical construct) is treated as if it were a concrete event or physical entity [21]. It occurs when something non-concrete is erroneously treated as if it were a tangible entity—such as the concept of the center of mass, a mere mathematical abstraction. In everyday language, this tendency is known as “nominalization” and is generally discouraged by linguists. Steven Pinker points out that nominalization is an undesirable habit of non-classical prose, where events are “thingified” by turning verbs and adjectives into nouns [65]. I contend that information belongs to this category—it is perhaps the most notable instance of nominalization in contemporary discourse.

Therefore, we might reformulate Peter Strawson’s famous statement: rather than “To suppose that, whenever we use a singular substantive, we are, or ought to be, using it to refer to something, is an ancient, but no longer a respectable, error” [66], we could assert that “To suppose that, whenever we use a mathematical quantitative expression, we are, or ought to be, using it to refer to something, is a recent and never respectable error.”

Similarly, William James employed the concepts of “vicious abstractionism” and “vicious intellectualism” extensively, particularly to criticize Immanuel Kant’s and Georg Wilhelm Friedrich Hegel’s idealist philosophies. James explains, “We conceive a concrete situation by singling out some salient or important feature in it, and classing it under that; then, instead of adding to its previous characters all the positive consequences which the new way of conceiving it may bring, we proceed to use our concept privatively; reducing the originally rich phenomenon to the naked suggestions of that name abstractly taken […] and acting as if all the other characters from out of which the concept is abstracted were expunged. Abstraction, functioning in this way, becomes a means of arrest far more than a means of advance in thought” [67].

In summary, I argue that information is a useful metaphor that does not necessitate any ontological commitment. According to Levy, informational language is “what we might call a liminal metaphor—one that operates near the threshold of the noticeable” [16]. Liminality is an essential characteristic of metaphorical language, particularly in scientific contexts. Importantly, denying that information has ontological substance does not imply that informational language or information-based models lack utility. On the contrary, they can indeed have explanatory and predictive power. In this context, Levy notes, “one can learn real-world facts by consulting fictions […] the fiction is constructed so that what is fictionally true corresponds to what is true simpliciter. Since the fiction employs a familiar set-up that makes it easier to handle, we use an indirect route and make fictional statements as a way of reasoning about the real world. To use a tracking metaphor, we use a fictional set-up to track non-fictional truths.” I believe there is strong justification to consider information as a useful fiction, as Levy proposes.

There is no substance called information; rather, it is a valuable model for addressing events that exhibit probabilistic correlations—such as genetic codes and phenotypes, inputs and outputs, real-world events and memory cells in computers, or external events and neural activity in the brain. Shannon’s original formula is an ingenious method for addressing the averaged and weighted probabilities of events. However, this should not lead to the reification of information as if it were an additional reality component; a deflationary stance is more prudent, especially given that Shannon’s information remains causally overdetermined by physical events.

In this paper, HR is a mathematical reformulation of H that does not presuppose an additional entity, such as information, thereby demonstrating that information is an epistemic construct that can be revisited in terms of probability. Naturally, I am not questioning the mathematical validity or the practical usefulness of bits, information processing, computational devices, and similar concepts. Instead, I challenge the metaphysical claim that these convenient models (beginning with Shannon’s original equation) imply ontological commitments. In contrast, HR indicates that, in principle, information-related equations can be restated purely in terms of probability theory without requiring further ontological or metaphysical assertions regarding the existence of information. Thus, I advocate for a deflationary perspective on information.

This conclusion has significant implications for consciousness research. Indeed, computational theories of consciousness typically assume robust ontological foundations for both computation and information. If consciousness is critically tied to information, as many would like to suggest, then information must exist fundamentally—a premise I call into question in this paper. In short, information is nothing more than an epistemic construct. Information is only a convenient way to refer to probability and does not fundamentally exist.

## Data Availability

No new data were created or analyzed in this study. Data sharing is not applicable to this article.
